# Transcriptome and Molecular Endocrinology Aspects of Epicardial Adipose Tissue in Cardiovascular Diseases: A Systematic Review and Meta-Analysis of Observational Studies

**DOI:** 10.1155/2015/926567

**Published:** 2015-11-09

**Authors:** Zhila Maghbooli, Arash Hossein-nezhad

**Affiliations:** ^1^Endocrinology and Metabolism Research Center, Endocrinology and Metabolism Clinical Sciences Institute, Tehran University of Medical Sciences, Tehran, Iran; ^2^Osteoporosis Research Center, Endocrinology and Metabolism Clinical Sciences Institute, Tehran University of Medical Sciences, 5th floor Shariati Hospital, North Kargar Avenue, Tehran 1411413137, Iran; ^3^Department of Medicine, Section of Endocrinology, Nutrition, and Diabetes, Vitamin D, Skin and Bone Research Laboratory, Boston University Medical Center, Boston, MA, USA

## Abstract

The objective of this study was to perform a systematic review of published literature on differentially expressed genes (DEGs) in human epicardial adipose tissue (EAT) to identify molecules associated with CVDs. A systematic literature search was conducted in PubMed, SCOPUS, and ISI Web of Science literature databases for papers published before October 2014 that addressed EAT genes and cardiovascular diseases (CVDs). We included original papers that had performed gene expressions in EAT of patients undergoing open-heart surgery. The Reporting Recommendations for Tumor Marker Prognostic Studies (PRIMARK) assessment tool was also used for methodological quality assessment. From the 180 papers identified by our initial search strategy, 40 studies met the inclusion criteria and presented DEGs in EAT samples from patients with and without CVDs. The included studies reported 42 DEGs identified through comparison of EAT-specific gene expression in patients with and without CVDs. Among the 42 DEGs, genes involved in regulating apoptosis had higher enrichment scores. Notably, interleukin-6 (IL-6) and tumor protein p53 (TP53) were the main hub genes in the network. The results suggest that regulation of apoptosis in EAT is critical for CVD development. Moreover, IL-6 and TP53 as hub genes could serve as biomarkers and therapeutic targets for CVDs.

## 1. Introduction

Among noncommunicable diseases, cardiovascular diseases (CVDs) are a major contributor to total global mortality and will continue to rise in the future. Thus, early detection of CVDs is critical for reducing the mortality and economic burden of this disease. Moreover, improving the understanding of the etiology associated with CVDs is highly important.

Over the last few decades, the pathophysiological concept of visceral adipose tissue has become an accepted indicator for CVD risk. Visceral adipose tissue is a metabolically active tissue that is highly involved in regulating different specific metabolic processes, including lipid metabolism, glucose homeostasis, angiogenesis, hemostasis, and blood pressure as well as the modulation of inflammation responses [1–5]. Recent evidence suggests that epicardial adipose tissue (EAT) as an index of cardiac visceral adiposity plays an essential role in cardiac morphology and function [6, 7]. EAT exists in the fat layer between the myocardium and visceral pericardium. Epicardial fat deposits are situated predominantly on the right-ventricular free wall and the left-ventricular apex but can also be directly located within the myocardium or around the coronary artery adventitia [8]. Anatomically, these fat deposits are not separated from the underlying myocardium. Studies have shown that EAT generates a variety of bioactive molecules, such as pro- and anti-inflammatory mediators and cytokines [9], which may significantly enhance paracrine effects on cardiac function or produce systemic effects that affect many physiological processes [10].

A growing body of research on EAT has focused mainly on target genes at the transcriptome level. These studies identified numerous differentially expressed genes in EAT that are associated with cardiovascular and metabolic risk factors. However, only a small number of these genes represent efficient biomarkers and therapeutic targets [10–12]. Nonetheless, molecular knowledge based on tissue-specific gene expression profiles is helpful for understanding many aspects of the pathogenic mechanisms of CVDs and cardiometabolic components as well as identifying tissue structures that may serve as potential targets for treating CVD.

In this study we conducted a systematic review of published gene expression studies on EAT that compared differentially expressed genes (DEGs) between patients with and without cardiometabolic risk factors for CVDs, especially coronary artery disease (CAD).

## 2. Methods

### 2.1. Search Strategy

Electronic searches in PubMed, Scopus, and ISI Web of Knowledge literature databases were performed by two investigators (Arash Hossein-nezhad and Zhila Maghbooli). The databases were searched for all relevant published studies published before October 18, 2014, using the search terms (TITLE-ABS-KEY (“epicardial adipose tissue”) OR TITLE-ABS-KEY (“epicardial fat”)) AND (TITLE-ABS-KEY (RT-qPCR) OR TITLE-ABS-KEY (real-time PCR) OR TITLE-ABS-KEY (real time PCR) OR TITLE-ABS-KEY (microarray) OR TITLE-ABS-KEY (gene expression profile) OR TITLE-ABS-KEY (gene expression) OR TITLE-ABS-KEY (transcriptome)). The first search was not restricted to human, animal, or experimental studies. Studies that analyzed EAT gene expression in humans were then selected.

### 2.2. Study Selection

The criteria for considering studies for inclusion were formalized in an inclusion criteria form (S1 Appendix a) (see Supplementary Material available online at http://dx.doi.org/10.1155/2015/926567). Two investigators (Arash Hossein-nezhad and Zhila Maghbooli) independently examined the titles and abstracts of the identified studies. If study eligibility was unclear from the abstract, then the full text of the paper was retrieved and independently evaluated by the assessors. Any disagreement about inclusion was resolved by discussion.

Eligible studies included in this review had the following criteria: human subjects undergoing open-heart surgery, differential gene expression, increase or decrease in differential expression or fold change in EAT, description of specific genes, and reliable definition of diseases and classification. Studies that did not meet one or more of the eligibility criteria were excluded. The studies were not limited to any language.

### 2.3. Data Extraction

Two investigators (Zhila Maghbooli and Arash Hossein-nezhad) independently extracted the data using a standardized form (S1 Appendix b). The form was pilot-tested on three studies to identify and reduce misinterpretations. The following topics were recorded from the included studies: author name, year of publication, study design, population (health/disease status, setting, sample size, age, and sex), phenotype (cardiovascular diseases: CAD, ischemic heart disease, and heart failure, and cardiometabolic risk factors: hypertension, insulin resistance, diabetes, and metabolic syndrome), clinical subtypes of interest, case and control definition, diagnostic criteria for CVDs and cardiometabolic risk factors, details on sampling and RNA preparation, and details of the statistical analysis used.

### 2.4. Quality Assessment

The methodological quality of included papers was assessed using the PRIMARK (Reporting Recommendations for Tumor Marker Prognostic Studies) assessment tool. We used 11 items included with the PRIMARK tool (S1 Appendix c). The study quality was based on the information reported in the papers and was simultaneously and independently assessed by two investigators (Zhila Maghbooli and Arash Hossein-nezhad) in the data extraction phase.

### 2.5. Differentially Expressed Gene (DEG) Analysis

From each selected paper, we extracted the published DEGs and selected the official gene symbol as the gene identifier. If only an alias name was given, then we used NCBI to obtain the official gene symbol provided by HUNC (HUGO Gene Nomenclature Committee). All included studies compared EAT gene expression between CVDs and/or cardiometabolic risk factors and control samples. Only those DEGs with a fold change value >1.5 and a *p* value < 0.05 were selected. For multiple testing corrections, we used the false discovery rate (FDR) [13, 14]. We performed further analyses on those genes that were identified as differentially expressed with a FDR < 0.05.

### 2.6. Enrichment and Functional Annotation

Enrichment and functional annotation analyses were performed using DAVID (the database for annotation, visualization, and integrated discovery, http://david.abcc.ncifcrf.gov/home.jsp), which is a web-accessible program aimed at systematically extracting biological meaning from large lists of genes [15]. In the present study, overrepresented Gene Ontology (GO) and functional annotation were detected with a value of *p* < 0.05 and an enrichment score ≥ 1. We selected the top ten significantly enriched GO terms.

### 2.7. Protein-Protein Interaction (PPI) Network Construction and Pathway Analyses

To demonstrate potential PPI networks, DEGs were mapped to the PPI data via the STRING database v.9.1 (Search Tool for the Retrieval of Interacting Genes) (http://www.stringdb.org/) [16]. The STRING database takes a meta-analysis approach toward protein-protein association information and identifies functional links between proteins. We constructed an extended network based on a high confidence score of 0.9, which implies that only interactions with a high level of confidence were extracted from the database and considered as valid links for the PPI network. Subcluster analysis was performed by *K*-means clustering. Indeed, STRING was previously used to identify significant KEGG (Kyoto Encyclopedia of Genes and Genomes) pathways (http://www.genome.jp/kegg/pathway.html).

## 3. Results

**Figure 1:**
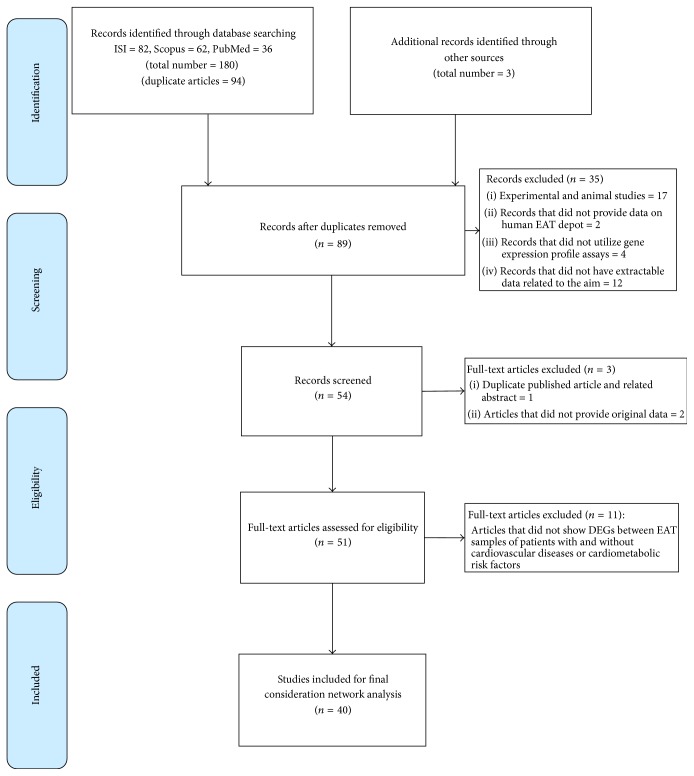
Flowchart of study selection process.

The systematic search identified 180 publications. Duplicate titles (*N* = 94) were removed and the title and abstract of the remaining 86 papers were screened based on inclusion and exclusion criteria. Of these, 35 studies did not meet the inclusion criteria as described: (i) 17 studies were not conducted in humans, (ii) 12 were clearly unrelated titles, (iii) 4 did not utilize gene expression profile assays, (iv) 2 did provide data on isolated adipocytes and stromal cells in EAT deposits, and (v) 3 studies were excluded for other reasons (one duplicate published article and related abstract; two studies did not provide original data). The full text of the remaining titles was then examined in more detail. Three new studies were identified from reference lists included in the eligible studies. All selected studies (*N* = 51) determined the mRNA expression in EAT obtained from patients undergoing elective heart surgery for either coronary artery bypass grafting or valve surgery. Of these, 40 studies reported DEGs in EAT samples from patients with and without CVDs [17–56] and 11 studies had a self-control design and only compared DEGs between epicardial and paired subcutaneous/abdominal adipose tissue samples [9, 57–65]. Finally, we included 40 studies in our analysis that compared EAT gene expression between patients with and without various CVDs, especially CAD and cardiometabolic risk factors (i.e., hypertension, insulin resistance, diabetes, and metabolic syndrome) (Figure 1).

### 3.1. Quality Assessment

Study characteristics and gene panels as described in the original papers were applied for quality assessment (Supplemental Table S1). Most studies adequately reported an acceptable definition of CVDs and cardiometabolic risk factors, the type and location of tissue sampling, RNA storage and isolation conditions, and the expression detection methods used. Most samples were stored in liquid nitrogen or at −80°C, and most studies used either RNeasy (Qiagen) or Trizol for RNA isolation. The majority of studies used a case-control design with an appropriate control group and adequately reported the characteristics of the case and control (e.g., age, sex, BMI, CAD severity, taking drugs, and cardiometabolic risk factors). Two studies did not report the results of DEGs despite collecting EAT from CAD and non-CAD patients and using a case-control study design [19, 44]. Several studies determined protein expression levels in EAT tissue in addition to the mRNA expression levels [17, 29, 32, 34, 39, 40, 46, 53], while some others investigated the correlation of EAT mRNA expression with CAD markers such as C-reactive protein (CRP) [32, 33]. The major obstacle in the quality assessment was the diversity in statistical procedures used to analyze the DEGs (normalization and analysis methods).

### 3.2. Identification of DEGs in EAT: Level of Association Evidence

All included studies used a candidate gene approach, except for three studies that used array-based gene expression methods and focused on only one or a few genes [25–27]. The included papers reported 112 genes as corresponding to cardiovascular dysfunction or metabolic syndrome (S2 Table). Among 112 genes, 32 were identified in at least two studies. The DEGs in patients with and without CVDs and/or cardiometabolic risk factors were identified. A total of 53 genes were selected as DEGs in EAT samples from patients with and without CVDs with a fold change >1.5 and *p* value < 0.05. All DEGs showed a consistent direction of expression change, except for PR Domain Containing 16 (PRDM16) and adrenomedullin (ADM) RNA expression levels, which had inconsistent changes in expression direction. Next, the resulting *p* values were corrected for multiple hypothesis testing by calculating the false discovery rate (FDR) with a cut-off of 0.05. There were forty-two genes that had a FDR < 0.05.

### 3.3. Gene Enrichment and Functional Annotation Analysis

To investigate the functional role of DEGs in EAT, the DEGs were mapped with DAVID. Among annotation clusters,* regulation of apoptosis, regulation of transcription factor activity*, and* regulation of systemic arterial blood pressure mediated by a chemical signal* had higher enrichment scores (enrichment score: 8.35, 6.51, 5.80, and 5.11, resp.). The top ten annotation clusters are shown in Table 1.

### 3.4. Protein-Protein Interaction Network and Pathway Analysis

**Figure 2:**
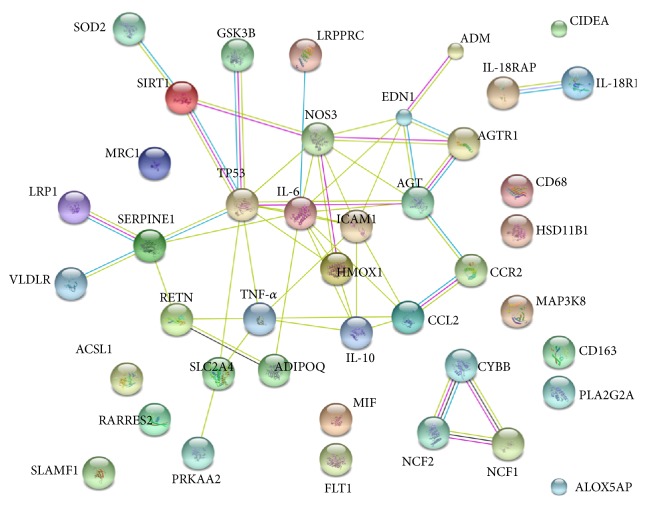
Protein-protein interaction (PPI) network constructed of differentially expressed genes (DEGs) identified in EAT samples. Forty-two DEGs were analyzed using the STRING database. IL-6 and TP53 were found to be the main hub genes.

**Figure 3:**
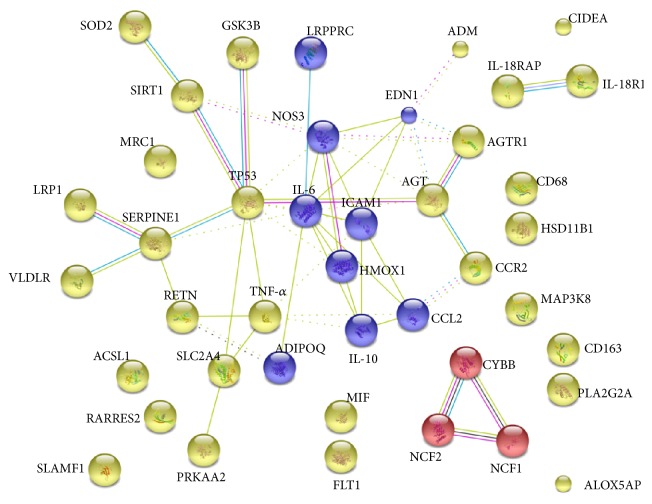
Subnetwork clusters identified from the PPI network. The resulting networks were clustered using *K*-means and confirmed IL-6 and TP53 as hub genes. Different line colors represent the types of evidence for the association.

To identify hub genes, a protein-protein interaction (PPI) network was constructed using the STRING database. In the network, each edge is examined by a score as the edge weight to quantify the interaction confidence. We projected DEGs with a FDR < 0.05 (42 genes) as inputs into the search tool (STRING) to determine the molecular network of interacting genes and obtain correlations with a high probability confidence score of ≥0.9 with a genome background. The results were significantly enriched in a network (*p* = 1.8*∗*10^−6^) with 52 interactions. Notably, interleukin-6 (IL-6) and tumor protein p53 (TP53) were the main nodes in the network (Figure 2). In addition, the network was further clustered using *K*-means clustering. Subsequent clustering identified at least three different functional clusters (Figure 3).

To gain insight into the biological processes of DEGs in EAT we used the STRING dataset. The first three biological processes were* response to external stimulus* (FDR = 1.51 × 10^−14^),* aging* (FDR = 1.85 × 10^−12^), and* response to activity* (FDR = 2.48 × 10^−10^) (Table 2).

To construct the PPI network, PPI data were obtained from the STRING database and significant enrichment of the DEGs in multiple KEGG terms was detected. The most significantly enriched pathways of DEGs were* HIF-1 signaling pathway* (FDR = 1.82 × 10^−7^),* TNF signaling pathway* (FDR = 1.82 × 10^−7^), and* cytokine-cytokine receptor interaction* (FDR = 3.56 × 10^−6^) (Table 3).

## 4. Discussion

Despite recent interventions to reduce cardiovascular risks, CVDs, especially CAD, remain the primary cause of death worldwide [66]. Emerging early detection biomarkers or nontraditional risk factors may have a potential role in providing new approaches to develop therapeutic targets for CAD and related risk factors. Epicardial adipose tissue (EAT) is an index of cardiac visceral adiposity and displays high metabolic activity [67]. EAT is known to secrete various bioactive proteins that contribute to local function of coronary vessels and the myocardium and/or have systemic endocrine effects on vascular tissue [68]. Both clinical and epidemiological studies have found an association between EAT and cardiometabolic risk factors and the progression of atherogenesis [48, 69], while EAT volume can act as a significant CAD predictor [48]. Therefore, identification of genes that are differentially expressed in EAT is critically important to understand the molecular mediators of CVD and to develop effective disease management strategies. Given that different gene expression studies used different candidate genes in EAT and different processing methods (statistical procedures), there is a lack of comprehensive information on the biological role of EAT in cardiovascular disease risk.

Our systematic review of studies focused on EAT and provides an overview of EAT DEGs in CVDs, especially CAD, and/or cardiometabolic risk factors. Here we extracted information from all of the included studies on DEGs in EAT samples and performed a meta-analysis based on gene-networks consisting of upregulated and downregulated genes in EAT to identify genes that may be involved in disease pathogenesis. When all 40 studies that generated data on mRNA expression in EAT were included, we identified 112 genes that could be related to CVDs and/or cardiometabolic risk factors. Our findings from the included studies showed that only 32 genes were identified in at least two studies, and of these, only seven genes were identified by more than three studies. The remainder of the genes appeared only once in the included studies. Furthermore, groups of genes that appeared once in a study might represent regulatory programs that are specific to disease mechanisms, such as pathways that are over- or underactive in CVD. Consequently, we included all DEGs in our network meta-analysis. Multiple test corrections showed that 42 genes were significantly differentially expressed in EAT from patients with and without CVDs and/or cardiometabolic risk factors. After removal of insignificantly expressed genes, functional annotation and enrichment analysis showed that the most important functions related to DEGs were* regulation of apoptosis* and* regulation of systemic arterial blood pressure*.

Apoptosis is a biological process of programmed cell death that is tightly regulated. Genes involved in regulating apoptotic processes that were identified in our clustering analysis included IL-6, CCL2, TNF-*α*, TP53, CIDEA, ADIPOQ, SIRT1, IL10, MIF, SOD2, HMOX1, AGT, GSK3B, and NOS3. All of these genes were previously reported to be apoptosis biomarkers. Dysregulated apoptosis signalling pathways have been shown to play an important role in the pathogenesis of CVDs [70]. Recent studies on the biochemical hallmarks of myocardial apoptosis and heart dysfunction suggest that excessive amounts of reactive oxygen species (ROS) in EAT can modulate apoptosis [17, 40, 71, 72]. Moreover, in CVD patients EAT produces higher levels of reactive oxygen species (ROS) relative to subcutaneous adipose tissue (SAT) [40]. Subsequent studies showed higher protein levels of IL-6, CCL2, and TNF-*α* in EAT compared to SAT in CAD patients [9], who were also found to have higher IL-6 and TNF-*α* and lower ADIPOQ in EAT compared with non-CAD control subjects [9, 20, 73]. Reactive oxygen species (ROS) have been proposed to be potential contributors to inflammatory pathways [41]. Thus, an imbalance between inflammatory and anti-inflammatory cytokines secreted by EAT may be strongly involved in the development and progression of CAD [48].

Dysregulation of processes involved in* systemic arterial blood pressure* has also been implicated in affecting heart function and the risk of developing CVDs [74, 75]. AGTR1, AGT (functional category: renin-angiotensin system), EDN1, NOS3 (functional category: endothelial integrity), and SOD2 (functional category: oxidation-reduction state), which participate in systemic arterial blood pressure regulation, are also EAT markers involved in the pathogenesis of hypertension and CVDs [20, 40, 41, 62, 76]. By mapping DEGs to the STRING database, we were able to construct a PPI network that identified IL-6 and TP53 as hub nodes.

Interleukin-6 is a cytokine that has both pro- and anti-inflammatory actions [77]. IL-6 is produced by several cell types in the cardiovascular system, including fibroblasts, monocytes, endothelial cells, and adipocytes, and has important roles in activating immune responses and metabolic balance as well as in maintaining cardiovascular homeostasis [78]. Visceral adipose tissue has been shown to release more IL-6 than SAT. As mentioned above, increased mRNA and protein expression levels of IL-6 were observed in EAT compared to paired SAT from patients with CAD [9, 58]. EAT IL-6 synthesis is thought to increase in response to hypoxia and subsequently alter local and/or systemic vascular inflammation that in turn increases the risk of CVDs. In fact, the proximity of EAT to coronary arteries and the absence of muscle fascia between the adipocytes and myocardial layer can cause upregulated IL-6 mRNA and protein levels to disturb ventricular function and increase the risk of CAD [9, 58, 78].

The implication of IL-6 in CVDs by the analyzed studies is consistent with conclusions drawn in a study by Eiras et al. [29], which observed that EAT IL-6 mRNA levels were significantly higher in CAD relative to non-CAD patients and that these elevated levels were positively correlated with the severity of CAD as well as an increased predicted risk of CAD. In addition, Eiras et al. showed that IL-6 was the only independently significant risk factor for CAD [29]. Taken together, these results suggest that IL-6 expression in EAT may have an important local effect on the extension of CAD.

In accordance with the present findings, Nair et al. [79] also showed that IL-6 occupies the center of a backbone network in a patient with CAD. Together these results suggest that IL-6 could be considered as a super-hub gene involved in heart dysfunction. Our findings confirm the hypothesis that IL-6 may locally affect heart function and thus could act as a biomarker of CVDs and as a predictor for disease onset.

TP53 is a protein that is well known for its association with cancer and is often described as “the guardian of the genome.” As a tumor suppressor and regulator of hundreds of target genes, TP53 can regulate numerous cellular processes, including cell cycle progression, apoptosis, cellular senescence, and DNA repair [80]. More recently, TP53 was implicated as a regulator of aging and thus could contribute to many aspects of aging and age-related diseases, such as cardiovascular and metabolic disorders [81, 82]. The actions of aging proteins like TP53 on CVD have been well studied [83] and suggest that TP53 and its cellular pathways contribute to disease pathogenesis.

In a recent follow-up study, Agra et al. showed higher TP53 mRNA expression levels in EAT than in paired SAT from patients undergoing cardiac surgery [17]. In addition, the authors demonstrated that EAT samples obtained from heart failure patients showed higher TP53 mRNA expression levels than those without heart failure and that TP53 expression was not associated with plasma adipokine levels. Moreover, patients who died during the follow-up period expressed lower levels of EAT TP53 relative to surviving patients [17]. These findings suggest that TP53 expression could be related to the inflammatory state present in heart failure patient EAT. The fact that TP53 expression was not associated with plasma adipokine levels also suggests the presence of local rather than systemic effects and regulation. Under normal conditions, TP53 induces expression of reactive oxygen scavenging genes that in turn provide protection to adipocytes from ROS. Conversely, during hypoxic, lipotoxic, or inflammatory situations, TP53 interacts with several downstream genes to induce apoptosis [38, 82].

In our meta-analysis of signaling pathways,* HIF-1 signaling pathway* and* TNF signaling pathway* headed the list of genes that are dysregulated in EAT from CVD patients. Indeed, the relationship of the HIF-1 and TNF signaling pathways to cardiometabolic risks has been extensively documented. Hypoxia inducible factor 1 (HIF-1) is a key regulator of oxygen homeostasis and mediates genomic responses to hypoxia. The activated HIF complex upregulates hypoxia inducible genes involved in cell proliferation, angiogenesis, glycolytic energy metabolism, and apoptosis [84]. The potential role of the HIF-1 transcriptional complex at a molecular and cellular level as well as functional responses in the heart to oxygen supply impairment has been broadly studied in the context of CVDs [85]. Under hypoxic conditions, transcriptional responses, such as TNF-*α*, IL-6, and TP53, are mediated by the HIF signaling pathway to promote angiogenesis and increase the oxygen supply to the heart [86, 87].

In recent years, it has emerged that TNF signaling plays a role in CAD pathogenesis, the development of atherosclerosis, heart failure, and the progression of myocardial disease [88]. Since TNF-*α* can induce apoptosis, its pathogenic effect on heart function may at least in part be due to its ability to induce cell death [89]. In contrast, there is also evidence to support a prosurvival role of TNF-*α* in the heart whereby TNF-*α* regulates adaptive responses to biomechanical stress [90]. Meanwhile, Shibasaki et al. demonstrated that higher expression of TNF-*α* and IL-6 in CAD patient EAT did not reflect the plasma levels of these markers [47], which suggests that the TNF signaling pathway in EAT may act locally via paracrine effects rather than circulating factors.

Our findings identified 40 DEGs and found that among these studies, there was a good overall agreement on the direction in which DEGs changed. An exception to this pattern was the data for PRDM16 and ADM RNA expression, which showed inconsistent directions of expression change. For ADM, Iacobellis et al. reported that ADM mRNA levels in EAT were significantly lower in patients with CAD than in those without CAD [34], while the mRNA expression levels of ADM in the EAT tissue were significantly higher in the CAD group than in the non-CAD group [47]. For PRDM16, Sacks et al. determined that PRDM16 expression in EAT was significantly lower in diabetes patients DM and higher in patients with metabolic syndrome (MetS) than control subjects [43]. However, all patients with DM and MetS had evidence of critical CAD. In another study performed by these authors, EAT PRDM16 expression was upregulated 1.84-fold in CAD patients compared to non-CAD patients [41].

There are limitations in our study. First, this systematic review focused on the role of mRNA expression in EAT samples rather than protein expression in the pathogenesis of CVD. However, a few studies determined the tissue protein expressions as well as their mRNA expression levels in EAT. Next, the statistical procedures used to analyze the DEGs of EAT, including normalization, are still unclear. Finally, due to diversity in statistical methods used for detecting differentially expressed genes, variation in patient classifications, and the lack of an appropriate control group in some studies, the evidence of that association remains weak.

## 5. Conclusion

In conclusion, we have shown complementary approaches that identified EAT transcriptomic information for patients with and without CVDs. We used network analysis and found that IL-6 and TP53 were the most important key genes related to cardiovascular risk which were expressed in EAT. These data suggest that IL-6 and TP53 in EAT could act to modulate heart function through HIF-1 and TNF signaling pathways. Confirmation of this link requires additional studies that will enhance our understanding of the pathogenesis role of EAT in cardiovascular diseases.

## Supplementary Material

Additional information about methods; study inclusion criteria, data extraction form (including description of specific genes, case definition and diagnosis, and differentially expressed Genes), and study quality assessment form (PRIMARK assessment tool) was described in Appendix S1. Characteristics of all studies included in the systematic review were listed in S1 Table. Differentially expressed genes in epicardial adipose tissue (EAT) of patients with cardiovascular diseases (CVDs) and/or cardiometabolic risk factors were listed S2 Table.

## Figures and Tables

**Table 1:** Gene enrichment and functional annotation analysis (top ten significantly enriched GO terms with a high count of DEGs in the EAT samples).

GO_id	Functional term	Enrichment score	*p* value	FDR *p* value
0043066	Negative regulation of apoptosis	8.356348318786127	3.95 × 10^−9^	6.51 × 10^−6^
0042981	Regulation of apoptosis	6.511095210354866	2.82 × 10^−7^	4.64 × 10^−4^
0051090	Regulation of transcription factor activity	5.808458303615781	5.23 × 10^−7^	8.62 × 10^−4^
0003044	Regulation of systemic arterial blood pressure mediated by a chemical signal	5.116925647742479	1.09 × 10^−6^	0.001802
0006873	Cellular ion homeostasis	4.704792812343122	1.12 × 10^−5^	0.018473
0051223	Regulation of protein transport	4.519156948056949	2.02 × 10^−5^	0.033305
0051091	Positive regulation of transcription factor activity	4.309175470478401	2.84 × 10^−5^	0.046671
0030335	Positive regulation of cell migration	3.766691351047639	1.33 × 10^−4^	0.219528
0030334	Regulation of cell migration	3.7019715102579265	1.32 × 10^−4^	0.21785
0016477	Cell migration	3.40004510443413	1.46 × 10^−4^	0.240623

**Table 2:** The GO biological processes enriched for the proteins present in the STRING protein network.

GO_id	Term	Genes in test set	Number of genes	*p* value	FDR *p* value
GO:0009605	Response to external stimulus	TP53; NCF2; SOD2; ADIPOQ; ICAM1; PRKAA2; NCF1; HMOX1; FLT1; MIF; AGT; ADM; RETN; ACSL1; RARRES2; SERPINE1; GSK3B; AGTR1; NOS3; CYBB; EDN1; IL-10; LRP1; MRC1; TNF-*α*; PLA2G2A	26	1.12 × 10^−18^	1.51 × 10^−14^

GO:0007568	Aging	IL-10; AGT; ADM; LRP1; IL-6; ICAM1; RETN; SIRT1; NCF2; SERPINE1; MIF; CCL2; EDN1;	13	2.75 × 10^−16^	1.85 × 10^−12^

GO:0014823	Response to activity	IL-10; NCF2; AGT; SOD2; ADIPOQ; TNF-*α*; EDN1; CCL2	8	5.53 × 10^−14^	2.48 × 10^−10^

GO:0071216	Cellular response to biotic stimulus	IL-10; SERPINE1; TNF-*α*; GSK3B; IL-6; ICAM1; CCL2; TP53; MRC1; NOS3	10	1.09 × 10^−13^	3.68 × 10^−10^

GO:0032496	Response to lipopolysaccharide	IL-10; ADM; ICAM1; MRC1; NOS3; NCF2; SERPINE1; TNF-*α*; CCL2; SOD2; EDN1	11	1.43 × 10^−12^	3.85 × 10^−9^

GO:0002237	Response to molecule of bacterial origin	IL-10; ADM; ICAM1; MRC1; NOS3; SERPINE1; NCF2; TNF-*α*; CCL2; SOD2; EDN1	11	2.33 × 10^−12^	5.23 × 10^−9^

GO:0070482	Response to oxygen levels	ADM; ICAM1; TP53; NCF2; SIRT1; TNF-*α*; CCL2; SOD2; SLC2A4; ADIPOQ; EDN1	11	3.42 × 10^−12^	6.57 × 10^−09^

GO:0023057	Negative regulation of signaling	TP53; NOS3; CIDEA; SOD2; ADIPOQ; EDN1; IL-10; LRP1; ICAM1; PRKAA2; TNF-*α*; HMOX1; MIF; AGT; ADM; IL-6; SERPINE1	17	4.07 × 10^−12^	6.79 × 10^−9^

GO:0010648	Negative regulation of cell communication	TP53; NOS3; CIDEA; SOD2; ADIPOQ; EDN1; IL-10; LRP1; ICAM1; PRKAA2; TNF-*α*; HMOX1; MIF; AGT; ADM; IL-6; SERPINE1	17	4.54 × 10^−12^	6.79 × 10^−9^

GO:0045428	Regulation of nitric oxide biosynthetic process	IL-10; AGT; SOD2; TNF-*α*; EDN1; IL-6; ICAM1	7	6.29 × 10^−12^	8.45 × 10^−9^

**Table 3:** Top ten enriched KEGG pathway of DEGs in EAT samples from patients with and without cardiovascular diseases.

GO id	Functional description KEGG ID	Genes in test set	Number of genes	*p* value	FDR *p* value
4066	HIF-1 signaling pathway	SERPINE1, HMOX1, IL-6, FLT1, CYBB, EDN1, NOS3	7	1.56 × 10^−9^	1.82 × 10^−7^
4668	TNF signaling pathway	MAP3K8, IL-18R1, TNF-*α*, IL-6, ICAM1, CCL2, EDN1	7	1.91 × 10^−9^	1.82 × 10^−7^
4060	Cytokine-cytokine receptor interaction	IL-18RAP, IL-10, IL-6, CCR2, IL-18R1, TNF-*α*, FLT1, CCL2	8	4.97 × 10^−8^	3.56 × 10^−6^
4068	FoxO signaling pathway	IL-10, SIRT1, IL-6, PRKAA2, SOD2, SLC2A4	6	1.59 × 10^−7^	8.1 × 10^−6^
5321	Inflammatory bowel disease (IBD)	IL-18RAP, IL-10, IL-18R1, TNF-*α*, IL-6	5	1.69 × 10^−7^	8.1 × 10^−6^
4920	Adipocytokine signaling pathway	ACSL1, SLC2A4, ADIPOQ, TNF-*α*, PRKAA2	5	2.51 × 10^−7^	1.03 × 10^−5^
5143	African trypanosomiasis	IL-6, IL-10, ICAM1, TNF-*α*	4	6.01 × 10^−7^	2.16 × 10^−5^
5323	Rheumatoid arthritis	TNF-*α*, IL-6, ICAM1, FLT1, CCL2	5	8.29 × 10^−7^	2.64 × 10^−5^
5142	Chagas disease (American trypanosomiasis)	IL-10, SERPINE1, TNF-*α*, IL-6, CCL2	5	1.52 × 10^−6^	4.36 × 10^−5^
5140	Leishmaniasis	IL-10, NCF2, TNF-*α*, NCF1	4	1.2 × 10^−5^	2.94 × 10^−4^
