# Supplementary material for: Transcriptome and Molecular Endocrinology Aspects of Epicardial Adipose Tissue in Cardiovascular Diseases: A Systematic Review and Meta-Analysis of Observational Studies
Source: Biomed Res Int. 2015 Nov 9;2015:926567. doi: 10.1155/2015/926567 (PMC4655271; doi:10.1155/2015/926567)
Supplement: Supplementary file 1 — Additional information about methods; study inclusion criteria, data extraction form (including description of specific genes, case definition and diagnosis, and differentially expressed Genes), and study quality assessment form (PRIMARK assessment tool) was described in Appendix S1. Characteristics of all studies included in the systematic review were listed in S1 Table. Differentially expressed genes in epicardial adipose tissue (EAT) of patients with cardiovascular diseases (CVDs) and/or cardiometabolic risk factors were listed S2 Table. [file 926567.f1.zip › appendix 1.docx]

**Appendix S1 a: Study inclusion criteria**

Studies that are considered eligible to be included in the review have the following features:

- Human study
- Relevant diseases
  - Coronary artery disease (CAD)
  - Cardiovascular diseases
  - Metabolic syndrome
  - Hypertension
  - Insulin resistance
  - Diabetes
- State the RNA gene expressions examined
- Describe the characteristics of the study patients
- Describe type of biological material used (including control samples) and methods of preservation and storage.
- Specify the assay method used and provide (or reference) a detailed protocol.

Studies considered not eligible and are therefore excluded from the review have one or more of the following features:

- Animal study
- Disease is not relevant

**Appendix S1 b: Data extraction form**

| **Study details** | ‐ ID (First author)  ‐ Year of publication  ‐ Study design  ‐ Assessments of validity |  |
| --- | --- | --- |
| **Gene expression** | ‐ Gene  ‐ detect expression  methods |  |
| **Population** | ‐ health/disease status  ‐ setting  ‐ sample size  ‐ age  ‐ sex  ‐ other important  classifications |  |
| **Tissue samples** | ‐ EAT |  |
| **Phenotypes** | ‐ case definition/diagnosis  ‐ clinical subtypes of  interest |  |
| **Findings** | ‐ Genotype data for  case and control  ‐ Measure of association  ‐ Interactions report |  |
| **Source of data** | ‐ All references linking  multiple publications  from the same study  ‐ State if data taken  direct from  publication, from  online database, from  correspondence with  study authors |  |

**Appendix S1 c: Quality Assessment form**

| **Introduction** |
| --- |
| 1. State the expression of genes examined, the study objectives, and any pre-specified hypotheses. |
| MATERIALS AND METHODS |
| Patients |
| 2. Describe the characteristics (e.g., disease stage or comorbidities) of the study patients,  Give the eligibility criteria, and the sources and methods of case ascertainment and control selection. |
|  |
| **Assay methods** |
| 3. Specify the assay method used and provide (or reference) a detailed protocol, including specific reagents or kits used, quality control procedures, reproducibility assessments, quantitation methods, and scoring and reporting protocols. Specify whether and how assays were performed blinded to the study endpoint. |
| Study design |
| 4. State the method of case selection, and whether stratification or matching (e.g., by stage of disease or age) was used. |
| 5. Precisely define all clinical endpoints examined. |
| 6. List all candidate variables initially examined. |
|  |
| **Statistical analysis methods** |
| 7. Specify all statistical methods, including details of any variable selection procedures and other model-building issues, how model assumptions were verified, and how missing data were handled. |
|  |
| **Results/Data** |
| 8. Report distributions of basic demographic characteristics (at least age and sex), standard (disease-specific) prognostic variables, and genetic markers, including numbers of missing values. |
| Analysis and presentation |
| 9. Show the association of the markers to outcome events. |
| 10. Present univariate analyses showing the relation between the marker and outcomes |
| 11. For key multivariable analyses, report estimated effects (e.g., hazard ratio) with confidence intervals for the marker and, at least for the final model, all other variables in the model. |
|  |
