# Supplementary material for: Transcriptome and Molecular Endocrinology Aspects of Epicardial Adipose Tissue in Cardiovascular Diseases: A Systematic Review and Meta-Analysis of Observational Studies
Source: Biomed Res Int. 2015 Nov 9;2015:926567. doi: 10.1155/2015/926567 (PMC4655271; doi:10.1155/2015/926567)
Supplement: Supplementary file 1 — Additional information about methods; study inclusion criteria, data extraction form (including description of specific genes, case definition and diagnosis, and differentially expressed Genes), and study quality assessment form (PRIMARK assessment tool) was described in Appendix S1. Characteristics of all studies included in the systematic review were listed in S1 Table. Differentially expressed genes in epicardial adipose tissue (EAT) of patients with cardiovascular diseases (CVDs) and/or cardiometabolic risk factors were listed S2 Table. [file 926567.f1.zip › S1 Table.docx]

**S1 Table: Characteristics of all studies included in the systematic review, as they were reported in the original papers**

| NO. | First author and year | Study groups | EAT Samples (Case/  Control) | Men (case%/  Control %) | Assessment method | Gene panel | Evaluation method | Ref |
| --- | --- | --- | --- | --- | --- | --- | --- | --- |
| 1 | Agra, 2014 | HF /NHF | 45/18 | 55.55/77.77 | - | **TP53, ADIPOQ** | Real-time | [[17](#_ENREF_16)] |
| 2 | Agra, 2014 | CAD / NCAD | 33/56 | 87.87/46.42 | Angiography | **S100A9** | Real-time | [[38](#_ENREF_37)] |
| 3 | Atalar, 2012 | CAD&MetS / NCAD&NMetS | 20/10 | 50.0/50.0 | IDF criteria | **HSD11B1, CD68+** | Real-time | [[18](#_ENREF_17)] |
| 4 | Baker, 2009 | CAD / NCAD | 16/18 | 93.0/62.0 | - | ADIPOQ, TNF-α,  CD-68, PTPRC, TLR1, TLR4 | Real-time | [[19](#_ENREF_18)] |
| 5 | Baker, 2006 | DM /NDM | 36/10 | -/- | - | **RETN**, **ADIPOQ**, **PTPRC**, **IL-6**, **AGT**, **TNF-α**, **SERPINE1** , **PLAT**, **LEP** | Real-time | [[20](#_ENREF_19)] |
| 6 | Bambace, 2014 | DM / NDM | 14/20 | 100/100 | ADA criteria | **ADIPOQ , CCL2,**  **CD-68** | Real-time | [[21](#_ENREF_20)] |
| 7 | Bambace, 2011 | CAD / NCAD | 11/10 | 100/100 | - | **ADIPOQ** | Real-time | [[22](#_ENREF_21)] |
| 8 | Cappellano, 2013 | CAD&IHD / NCAD&NIHD | 4/4 | 83.33/80.0 | Angiography | **CNR1, CNR2** | Real-time | [[23](#_ENREF_22)] |
| 9 | Castro, 2012 | IR / NIR | 13/22 | 69.23/72.27 | HOMA-IR | **NR3C1**, **HSD11B1** | Real-time | [[24](#_ENREF_23)] |
| 10 | Dozio, 2014 | CAD / NCAD | 63/19 | 95.24/84.21 | Angiography | **IL15, IL15RA** | Micro-array | [[26](#_ENREF_25)] |
| 11 | Dozio, 2012 | CAD / NCAD | 23/9 | 95.65/77.78 | Angiography | **IL18RAP, IL18,**  **IL18R 1** | Micro-array | [[25](#_ENREF_24)] |
| 12 | Dutour, 2010 | CAD / NCAD | 30/30 | - | Angiography | **PLA2G2A** | Micro-array/  real-time | [[2](#_ENREF_26)7] |
| 13 | Eiras, 2010 | CAD / NCAD | 11/11 | 90.9/45.45 | Angiography | **CCL2, IL10, TNF-α** | Real-time | [[28](#_ENREF_27)] |
| 14 | Eiras, 2008 | CAD / NCAD | 58/34 | 82.75/52.94 | Angiography | **ADIPOQ , IL-6** | Real-time | [[29](#_ENREF_28)] |
| 15 | Fain, 2008 | DM&CAD / MetS&CAD | 14/13 | 50.0/53.84 | Angiography, ADA and ATP III criteria | **ITLN1, NAMPT** | Real-time | [[30](#_ENREF_29)] |
| 16 | Fandino Vaquero, 2014 | CAD / NCAD | 27/28 | 78.0/43.0 | Angiography | **ORM1** | Real-time | [[31](#_ENREF_30)] |
| 17 | Gao, 2011 | CAD / NCAD | 37/16 | 81.1/75.0 | Angiography | **RARRES2, CMKLR1, ADIPOQ , TNF-α** | Real-time | [[32](#_ENREF_31)] |
| 18 | Gormez, 2011 | CAD&MetS / NCAD&NMetS | 37/23 | 56.8/47.8 | ATP III criteria | **ADIPOQ , TNF-α, LEP** | Real-time | [[3](#_ENREF_32)3] |
| 19 | Iacobellis , 2009 | CAD / NCAD | 12/10 | 75.0/30.0 | Angiography | **ADM** | Real-time | [[3](#_ENREF_33)4] |
| 20 | Iglesias , 2006 | IHD / NIHD | 34/17 | - | - | **ADIPOQ , LEP** | Real-time | [[35](#_ENREF_34)] |
| 21 | Jaffer , 2012 | CAD / NCAD | 16/14 | 87.5/64.28 | Angiography | **LIPE**, **LPL**, **PNPLA2**, **ABHD5**,**PLIN1**, **PLIN5**, **ANGPTL4**,  IL-6, ADIPOQ, LEP | Real-time | [[3](#_ENREF_35)6] |
| 22 | Langheim, 2010 | CAD / NCAD | 34/23 | 100/100 | Angiography | **ADIPOQ** , **LEP, RETN, IL6, IL10, CCL2, SERPIN1, CD68, TNF-α, MIF** | Real-time | [[3](#_ENREF_36)7] |
| 23 | Nasarre, 2014 | DM / NDM | 54/22 | 79.63/77.27 | - | **LRP1, VLDLR** | Real-time | [[39](#_ENREF_38)] |
| 24 | Rodino-Janeiro, 2011 | CAD / NCAD | 17/20 | 76.5/50.0 | Angiography | **CAT, CYBB, NOX4, SYBA, NOX5, AGER** | Real-time | [40] |
| 25 | Sacks, 2011 | CAD / NCAD | 16/20 | 41.66/50.0 | Angiography | **IL8, SLAMF1, MAP3K8, ICAM1, CD163, CCL2, MRC1, CCR2, LCN2, CD68, ALOX5, ALOX5AP, SERPINE1, NFKB1, CCL5, TNF-α, NGF, GPX3, CYBB, NCF1, HMOX1, NOS3, NCF2, SOD2, AGT, AGTR1, MEOX2, FLT1, EDN1, SIRT1, PRDM16, PRKAA2, HSD11B1, ACSL1, CIDEA, LEP, FABP4, GSK3B, LRPPRC, PPARGC1A, ADIPOQ, RBP4, IL6, LPL, A1BG, ADM, HP, APLN, PTGDS, LIPE, THBS1, PLIN1, UCP2, VEGFA, KDR, SPP1, SCD, INHA, SMAD2, DIO2, SOCS1, CEBPB, PPARG, AZGP1, CD14, CFD, PLA2G2A, SPP1, CAT** | Real-time | [[41](#_ENREF_40)] |
| 26 | Sacks, 2009 | MetS&CAD+ DM&CAD/  NCAD&NDM&NMetS | 44/6 | 59.09/16.66 | Angiography | **UCP1, PRDM16, PPARGC1A** | Real-time | [[43](#_ENREF_42)] |
| 27 | Sacks, 2011 | CAD&MetS&DM / NCAD&NMetS&  NDM | 44/12 | -/- | Angiography | **IL10**, **IL1B**, **IL1RN**, **PPARG** | Real-time | [[42](#_ENREF_41)] |
| 28 | Salgado-somoza, 2012 | CAD / NCAD | 16/19 | 72.22/52.63 | Angiography | **RBP4**, **SLC2A4** | Real-time | [[46](#_ENREF_45)] |
| 29 | Salgado-Somoza, 2010 | CAD / NCAD | 12/12 | -/- | - | **CAT, GSTP1, P4HB, PGAM1** | Real-time | [[45](#_ENREF_44)] |
| 30 | Salgrdo-Somoza, 2012 | CAD / NCAD | 17/15 | 94.73/52.63 | Angiography | APOA1 | Real-time | [[44](#_ENREF_43)] |
| 31 | Shibasaki, 2010 | CAD / NCAD | 20/14 | 55.0/28.57 | Angiography | **IL6, IL1B, TNF-α, CCL2, PPARG, LEP, NPR1, NPR3** | Real-time | [[4](#_ENREF_46)7] |
| 32 | Shimabukuro, 2013 | CAD / NCAD | 50/50 | 86.0/54.0 | Angiography | **NLRP3**, **IL1B**, TNF- α, IL18, **ADIPOQ** , IL-6, CCL2, CCL5, TGFB1, IL1RN, ARG1, CCL18 | Real-time | [[4](#_ENREF_47)8] |
| 33 | Silaghi, 2012 | CAD / NCAD | 15/12 | 66.66/58.33 | Angiography | **NR3C1** | Real-time | [[50](#_ENREF_49)] |
| 34 | Silaghi, 2007 | CAD / NCAD | 12/10 | 58.33/50.0 | Angiography | **ADM**, **CALCRL**, **RAMP2, RAMP3, HSD11B1** | Real-time | [[49](#_ENREF_48)] |
| 35 | Teijeira-Fernandez, 2008 | HT / NHT | 84/32 | 63.0/78.0 | Angiography/  BP>140/90mm Hg | **ADIPOQ** | Real-time | [[5](#_ENREF_51)2] |
| 36 | Teijeira-Fernandez, 2011 | MetS / NMetS | 29/17 | 76.0/53.0 | ATP III criteria | **ADIPOQ** | Real-time | [[5](#_ENREF_50)1] |
| 37 | Teijeira-Fernandez, 2010 | DM / NDM | 45/75 | 64.0/71.0 | Angiography | **ADIPOQ , LEP** | Real-time | [[53](#_ENREF_52)] |
| 38 | Teijeira-Fernandez , 2012 | CV events /  NCV events | 34/103 | 76.0/67.0 | Angiography | **ADIPOQ , LEP** | Real-time | [[54](#_ENREF_53)] |
| 39 | Vural, 2008 | MetS / NMetS | 10/4 | 50.0/50.0 | ATP III criteria | **FABP4** | Real-time | [[5](#_ENREF_54)5] |
| 40 | Zhou, 2011 | CAD / NCAD | 9/11 | -/- | Angiography | **ADIPOQ , IL6, TNF-α, TLR4** | Real-time | [[5](#_ENREF_55)6] |

All genes included in the analysis were shown in bold format. Not all studies reported differentially expressed mRNAs of their gene panel in classified groups. CAD: Coronary Artery disease; NCAD: Non- Coronary Artery disease; CV events: Cardiovascular events; NCV events: Non- Cardiovascular events (CV events were defined as coronary acute syndrome, need for revascularization, heart failure, CV death or stroke); DM: type 2 Diabetes Mellitus; NDM: Non- type 2 Diabetes Mellitus; IR: Insulin Resistance; NIR: Non- Insulin Resistance; IHD: Ischemic Heart Disease; NIHD: Non- Ischemic Heart Disease; HF: Heart Failure; NHF: Non-Heart Failure; MetS: Metabolic Syndrome; NMetS: Non- Metabolic Syndrome; ATP III: Adult Treatment Panel III; BP: Blood Pressure; HOMA-IR: Homeostatic Model Assessment-Insulin Resistance; IDF: International Diabetes Federation; ADA: American Diabetes Association
