# Supplementary material for: Transcriptome and Molecular Endocrinology Aspects of Epicardial Adipose Tissue in Cardiovascular Diseases: A Systematic Review and Meta-Analysis of Observational Studies
Source: Biomed Res Int. 2015 Nov 9;2015:926567. doi: 10.1155/2015/926567 (PMC4655271; doi:10.1155/2015/926567)
Supplement: Supplementary file 1 — Additional information about methods; study inclusion criteria, data extraction form (including description of specific genes, case definition and diagnosis, and differentially expressed Genes), and study quality assessment form (PRIMARK assessment tool) was described in Appendix S1. Characteristics of all studies included in the systematic review were listed in S1 Table. Differentially expressed genes in epicardial adipose tissue (EAT) of patients with cardiovascular diseases (CVDs) and/or cardiometabolic risk factors were listed S2 Table. [file 926567.f1.zip › S2 Table.docx]

**S2 Table: Differentially expressed genes in epicardial adipose tissue (EAT) of patients with cardiovascular diseases (CVDs) and/ or cardiometabolic risk factors**

| Gen name* | Gene  ID* | Description* | mRNA Expression in CVDs and/or cardiometabolic risk factors compare to control subjects** |
| --- | --- | --- | --- |
| A1BG | 1 | alpha-1-B glycoprotein | NS |
| ABHD5 | 51099 | abhydrolase domain containing 5 | NS |
| ACSL1 | 2180 | acyl-CoA synthetase long-chain family member 1 | Up |
| ADIPOQ | 9370 | adiponectin, C1Q and collagen domain containing | Down |
| ADM | 133 | adrenomedullin | Up/Down |
| AGER | 177 | advanced glycosylation end product-specific receptor | NS |
| AGT | 183 | angiotensinogen (serpin peptidase inhibitor, clade A, member 8) | Up |
| AGTR1 | 185 | angiotensin II receptor, type 1 | Up |
| ALOX5 | 240 | arachidonate 5-lipoxygenase | NS |
| ALOX5AP | 241 | arachidonate 5-lipoxygenase-activating protein | Up |
| ANGPTL4 | 51129 | angiopoietin-like 4 | NS |
| APLN | 8862 | apelin | NS |
| AZGP1 | 563 | alpha-2-glycoprotein 1, zinc-binding pseudogene 1; alpha-2-glycoprotein 1, zinc-binding | NS |
| CALCRL | 10203 | calcitonin receptor-like | NS |
| CAT | 847 | catalase | NS |
| CCL2 | 6347 | chemokine (C-C motif) ligand 2 | Up |
| CCL5 | 6352 | chemokine (C-C motif) ligand 5 | NS |
| CCR2 | 729230 | chemokine (C-C motif) receptor 2 | Up |
| CD14 | 929 | CD14 molecule | NS |
| CD163 | 9332 | CD163 molecule | Up |
| CD68 | 968 | CD68 molecule | Up |
| CEBPB | 1051 | CCAAT/enhancer binding protein (C/EBP), beta | NS |
| CFD | 1675 | complement factor D (adipsin) | NS |
| CIDEA | 1149 | cell death-inducing DFFA-like  effector a | Up |
| CMKLR1 | 1240 | chemokine-like receptor 1 | NS |
| CNR1 | 1268 | cannabinoid receptor 1 (brain) | NS |
| CNR2 | 1269 | cannabinoid receptor 2 (macrophage) | NS |
| CYBA | 1535 | cytochrome b-245, alpha polypeptide | NS |
| CYBB | 1536 | cytochrome b-245, beta polypeptide | Up |
| DIO2 | 1734 | deiodinase, iodothyronine, type II | NS |
| EDN1 | 1906 | endothelin 1 | Up |
| FABP4 | 2167 | fatty acid binding protein 4, adipocyte | NS |
| FLT1 | 2321 | fms-related tyrosine kinase 1 (vascular endothelial growth factor/vascular permeability factor receptor) | Up |
| GPX3 | 2878 | glutathione peroxidase 3 (plasma) | NS |
| GSK3B | 2932 | glycogen synthase kinase 3 beta | Up |
| GSTP1 | 2950 | glutathione S-transferase pi 1 | NS |
| HMOX1 | 3162 | heme oxygenase (decycling) 1 | Up |
| HP | 3240 | haptoglobin-related protein; haptoglobin | NS |
| HSD11B1 | 3290 | hydroxysteroid (11-beta) dehydrogenase 1 | Up |
| ICAM1 | 3383 | intercellular adhesion molecule 1 | Up |
| IL1B | 3553 | interleukin 1, beta | NS |
| IL10 | 3586 | interleukin 10 | Up |
| IL15 | 3600 | Interleukin 15 | NS |
| IL15RA | 3601 | interleukin 15 receptor, alpha | NS |
| IL18 | 3606 | interleukin 18 (interferon-gamma-inducing factor) | NS |
| IL18R1 | 8809 | interleukin 18 receptor 1 | Up |
| IL18RAP | 8807 | interleukin 18 receptor accessory protein | Up |
| IL1RN | 3557 | interleukin 1 receptor antagonist | NS |
| IL6 | 3569 | interleukin 6 (interferon, beta 2) | Up |
| IL8 | 3576 | interleukin 8 | NS |
| INHA | 3623 | inhibin, alpha | NS |
| ITLN1 | 55600 | intelectin 1 (galactofuranose binding) | NS |
| KDR | 3791 | kinase insert domain receptor (a type III receptor tyrosine kinase) | NS |
| LCN2 | 3934 | lipocalin 2 | NS |
| LIPE | 3991 | lipase, Hormone-Sensitive | NS |
| LPL | 4023 | lipoprotein lipase | NS |
| LRP1 | 4043 | low density lipoprotein-related protein 1 (alpha-2-macroglobulin receptor) | Up |
| LRPPRC | 10128 | leucine-rich PPR-motif containing | Up |
| MAP3K8 | 1326 | mitogen-activated protein kinase kinase kinase 8 | Up |
| MEOX2 | 4223 | mesenchyme homeobox 2 | **NS** |
| MIF | 4282 | macrophage migration inhibitory factor (glycosylation-inhibiting factor) | Down |
| MRC1 | 4360 | mannose receptor, C type 1 | Up |
| NAMPT | 10135 | nicotinamide phosphoribosyltransferase | NS |
| NCF1 | 654817 | neutrophil cytosolic factor 1; neutrophil cytosolic factor 1C pseudogene | Up |
| NCF2 | 4688 | neutrophil cytosolic factor 2 | Up |
| NFKB1 | 4790 | nuclear factor of kappa light polypeptide gene enhancer in B-cells 1 | NS |
| NGF | 4803 | nerve growth factor (beta polypeptide) | NS |
| NLRP3 | 114548 | NLR family, pyrin domain containing 3 | NS |
| NOS3 | 4846 | nitric oxide synthase 3 (endothelial cell) | Up |
| NOX4 | 50507 | NADPH oxidase 4 | NS |
| NOX5 | 79400 | NADPH oxidase, EF-hand calcium binding domain 5 | NS |
| NPR1 | 4881 | natriuretic peptide receptor 1 | **NS** |
| NPR3 | 4883 | natriuretic peptide receptor 3 | NS |
| NR3C1 | 2908 | nuclear receptor subfamily 3, group C, member 1 (glucocorticoid receptor) | NS |
| ORM1 | 5004 | orosomucoid 1 | NS |
| P4HB | 5034 | prolyl 4-hydroxylase, beta polypeptide | NS |
| PGAM1 | 5223 | phosphoglycerate mutase 1 (brain) | NS |
| PLA2G2A | 5320 | phospholipase A2, group IIA (platelets, synovial fluid) | Up |
| PLIN5 | 440503 | lipid storage droplet protein 5 | NS |
| PLIN1 | 5346 | perilipin 1 | **NS** |
| PNPLA2 | 57104 | patatin-like phospholipase domain containing 2 | NS |
| PPARG | 5468 | peroxisome proliferator-activated receptor gamma | NS |
| PPARGC1A | 10891 | peroxisome proliferator-activated receptor gamma, coactivator 1 alpha | NS |
| PRDM16 | 63976 | PR domain containing 16 | NS |
| PRKAA2 | 5563 | protein kinase, AMP-activated, alpha 2 catalytic subunit | Up |
| PTGDS | 27306 | prostaglandin D2 synthase, hematopoietic; prostaglandin D2 synthase 21kDa (brain) | NS |
| PTPRC | 5787 | protein tyrosine phosphatase, receptor type, C | NS |
| RAMP2 | 10266 | receptor (G protein-coupled) activity modifying protein 2 | NS |
| RAMP3 | 10268 | receptor (G protein-coupled) activity modifying protein 3 | NS |
| RARRES2 | 5919 | retinoic acid receptor responder (tazarotene induced) 2 | Up |
| RBP4 | 5950 | retinol binding protein 4, plasma | NS |
| RETN | 56729 | resistin | Up |
| S100A9 | 6280 | S100 calcium binding protein A9 | NS |
| SCD | 6319 | stearoyl-CoA desaturase (delta-9-desaturase) | **NS** |
| SERPINE1 | 5054 | serpin peptidase inhibitor, clade E (nexin, plasminogen activator inhibitor type 1), member 1 | Up |
| SYBA | 1536 | cytochrome b-245, beta polypeptide | NS |
| SIRT1 | 23411 | sirtuin (silent mating type information regulation 2 homolog) 1 (S. cerevisiae) | Up |
| SLAMF1 | 6504 | signaling lymphocytic activation molecule family member 1 | Up |
| SLC2A4 | 6517 | solute carrier family 2 (facilitated glucose transporter), member 4 | Down |
| SMAD2 | 4087 | SMAD family member 2 | NS |
| SOCS1 | 8651 | suppressor of cytokine signaling 1 | NS |
| SOD2 | 6648 | superoxide dismutase 2, mitochondrial | Up |
| SPP1 | 6696 | secreted phosphoprotein 1 | NS |
| THBS1 | 7057 | thrombospondin 1 | NS |
| TLR4 | 7099 | toll-like receptor 4 | NS |
| TNFA | 7124 | Tumor necrosis factor-alpha | Up |
| TNFRSF11B | 4982 | tumor necrosis factor receptor superfamily, member 11b | NS |
| TP53 | 7157 | tumor protein p53 | Up |
| UCP1 | 7350 | uncoupling protein 1 (mitochondrial, proton carrier) | NS |
| UCP2 | 7351 | uncoupling protein 2 (mitochondrial, proton carrier) | NS |
| VEGFA | 7422 | vascular endothelial growth factor A | NS |
| VLDLR | 7436 | very low density lipoprotein receptor | Up |

A total of 53 genes were selected as DEGs in EAT samples from patients with and without cardiovascular diseases and/or cardio metabolic risk with a fold-change >1.5 and/or p-value<0.05. For multiple testing corrections, the false discovery rate (FDR) was used. The cut off for FDR was 0.05. There were forty-two genes which had FDR<0.05. All differentially expressed genes were with consistent direction except two genes: ADM and PRDM16 RNA expressions (Up/Down).

*Gene name, gene ID and gene description are based on HGNC data base of human gene names (HUGO gene nomenclature committee)

** The significance cut off level: FDR<0.05; significantly Up-regulated: Up; significantly Down-regulated: Down; Non-Significant: NS
